# Supplementary material for: Mutant p53 Directs PARP to Regulate Replication Stress and Drive Breast Cancer Metastasis
Source: bioRxiv. 2026 Mar 28:2026.03.26.713220. Preprint. [Version 1] doi: 10.64898/2026.03.26.713220 (PMC13041838; doi:10.64898/2026.03.26.713220)
Supplement: Supplement 1 [file media-1.pdf]

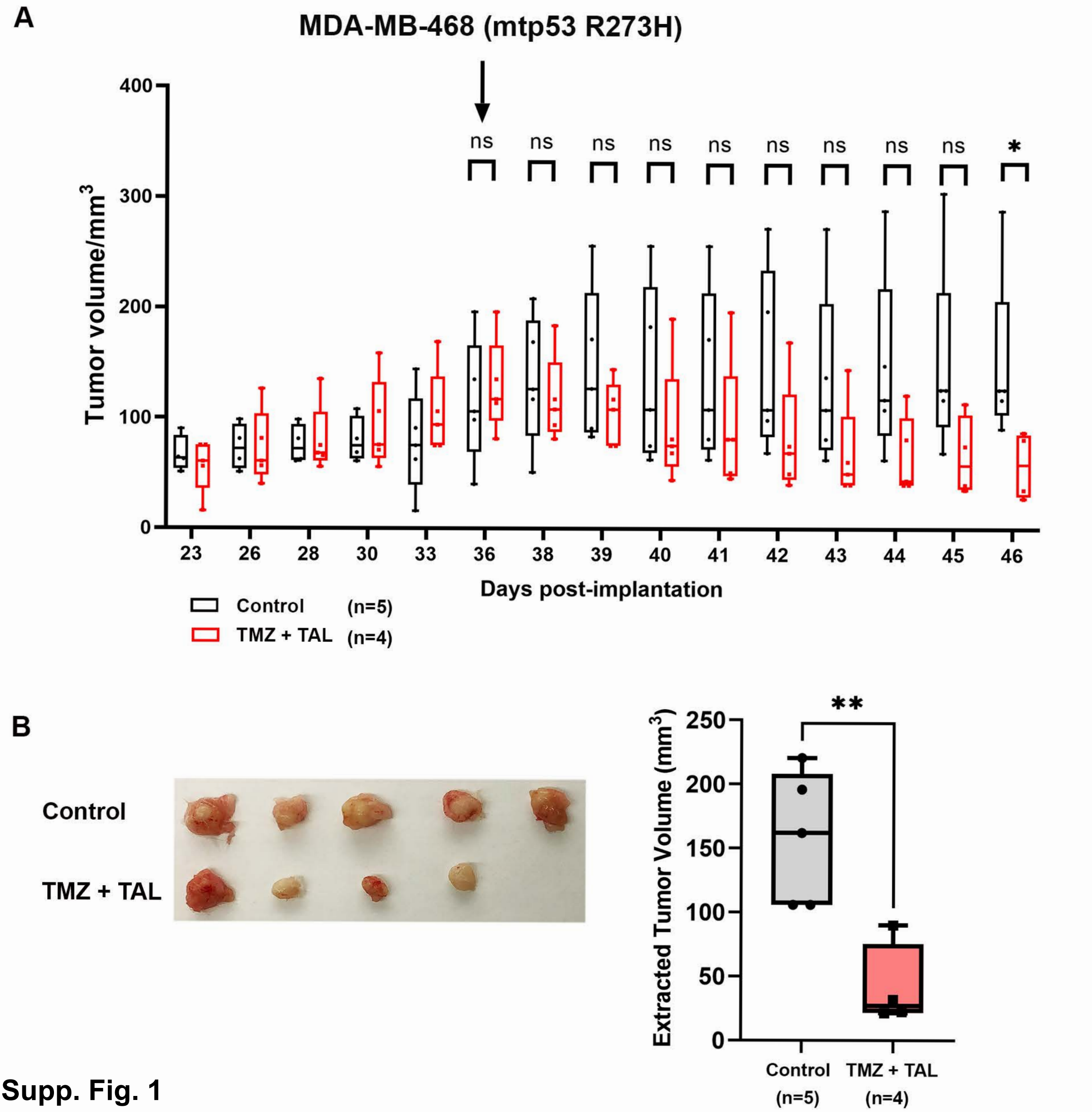
A
MDA-MB-468 (mtp53 R273H)
Tumor volume/mm3
400
300
200
100
0
ns
*
23
26
28
30
33
36
38
39
40
41
42
43
44
45
46
Days post-implantation
Control (n=5)
TMZ + TAL (n=4)
B
Control
TMZ + TAL
Extracted Tumor Volume (mm3)
250
200
150
100
50
0
**
Control (n=5)
TMZ + TAL (n=4)

Supp. Fig. 1

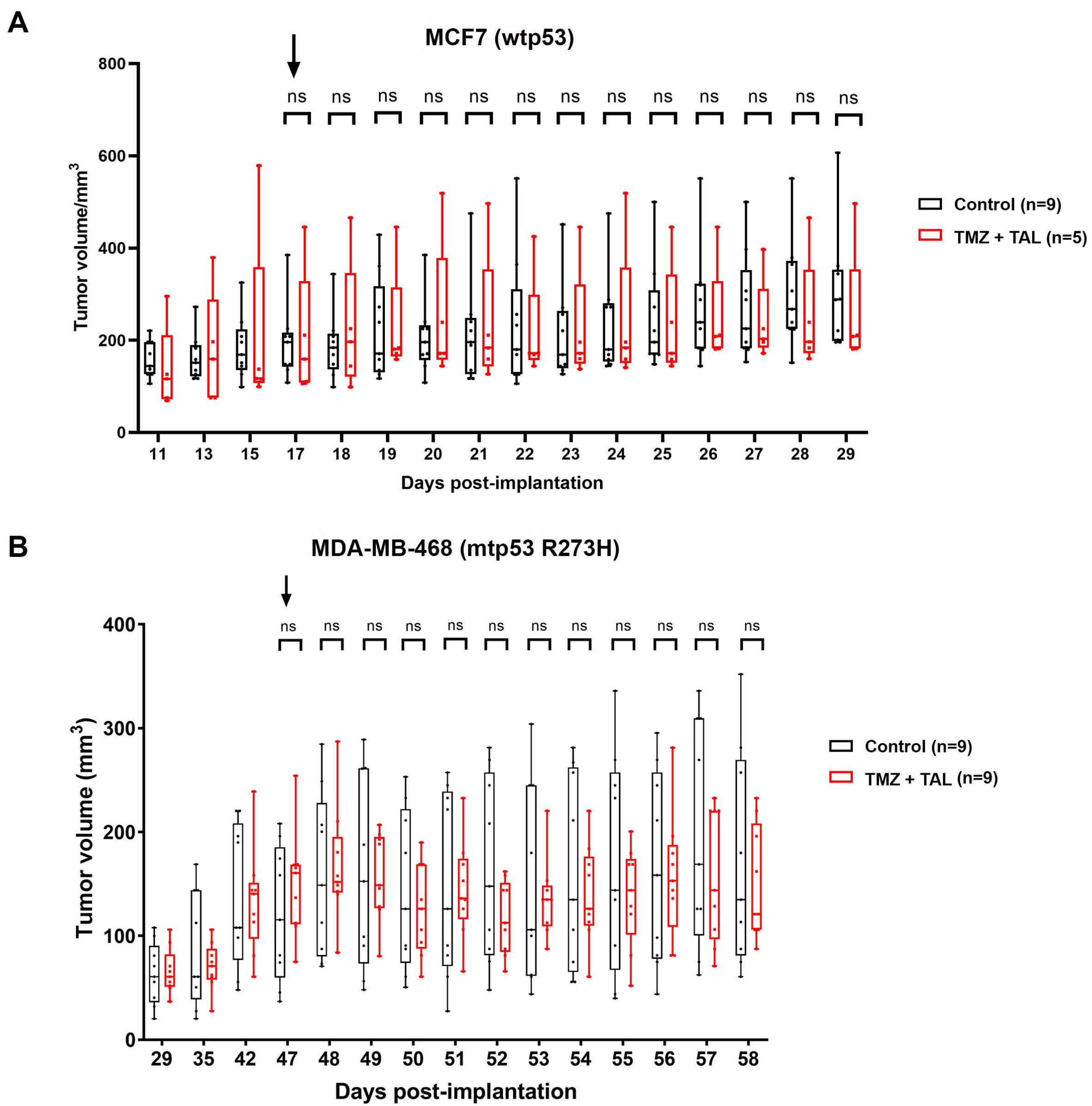

A
MCF7 (wtp53)
ns
Tumor volume/mm3
800
600
400
200
0
11 13 15 17 18 19 20 21 22 23 24 25 26 27 28 29
Days post-implantation
Control (n=9)
TMZ + TAL (n=5)
B
MDA-MB-468 (mtp53 R273H)
ns
Tumor volume (mm3)
400
300
200
100
0
29 35 42 47 48 49 50 51 52 53 54 55 56 57 58
Days post-implantation
Control (n=9)
TMZ + TAL (n=9)

Supp. Fig. 2

A

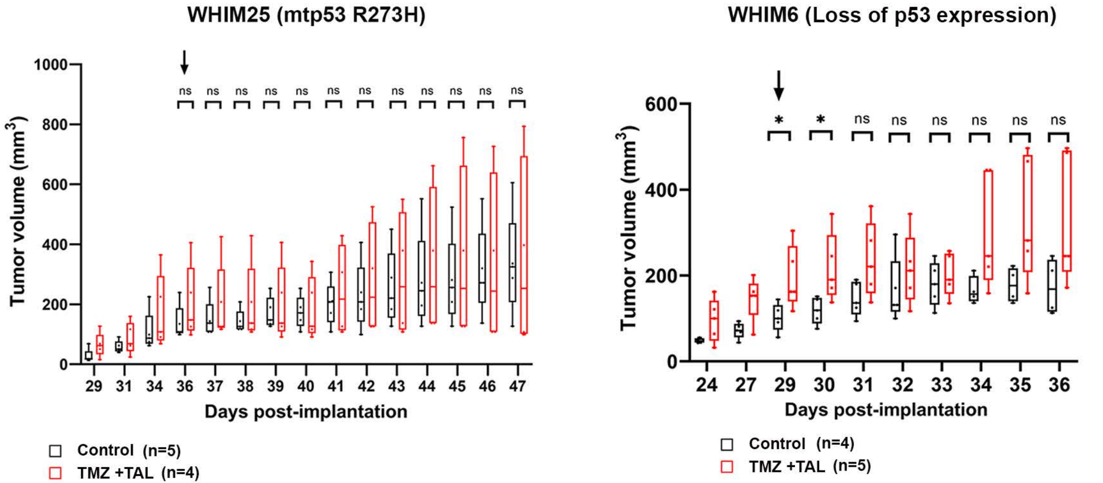
WHIM25 (mtp53 R273H)
Tumor volume (mm^3)
1000
800
600
400
200
0
ns ns ns ns ns ns ns ns ns ns ns ns
29 31 34 36 37 38 39 40 41 42 43 44 45 46 47
Days post-implantation
Control (n=5)
TMZ +TAL (n=4)
WHIM6 (Loss of p53 expression)
Tumor volume (mm^3)
600
400
200
0
* * ns ns ns ns ns ns
24 27 29 30 31 32 33 34 35 36
Days post-implantation
Control (n=4)
TMZ +TAL (n=5)

B

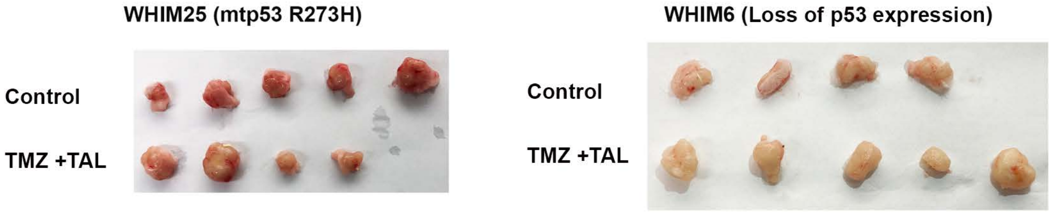
WHIM25 (mtp53 R273H)
Control
TMZ +TAL
WHIM6 (Loss of p53 expression)
Control
TMZ +TAL

Supp. Fig. 3

mtp53 R273H

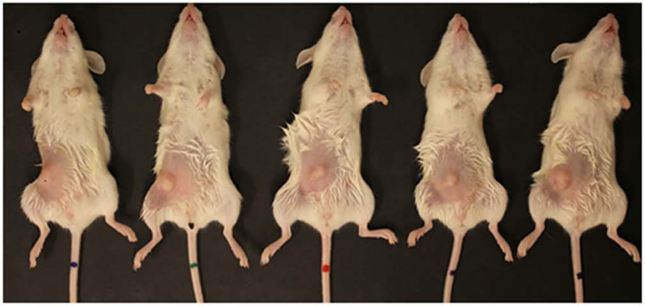

mtp53 R273HΔ347-393

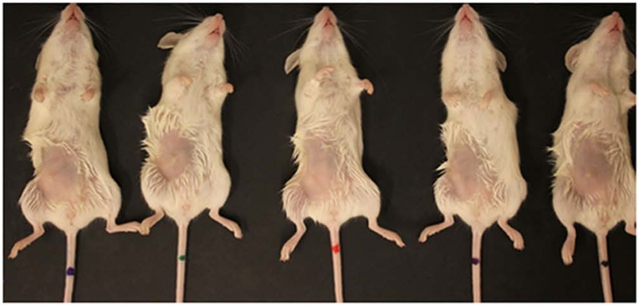

mtp53 R273Hfs387

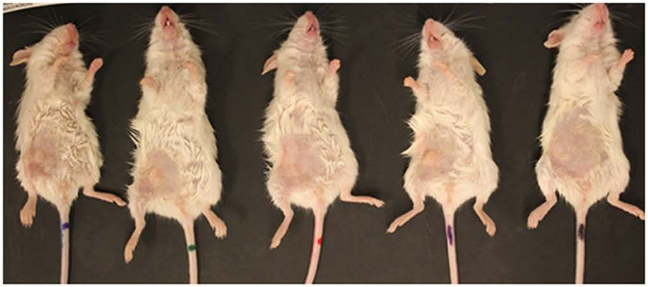

Supp. Fig. 4

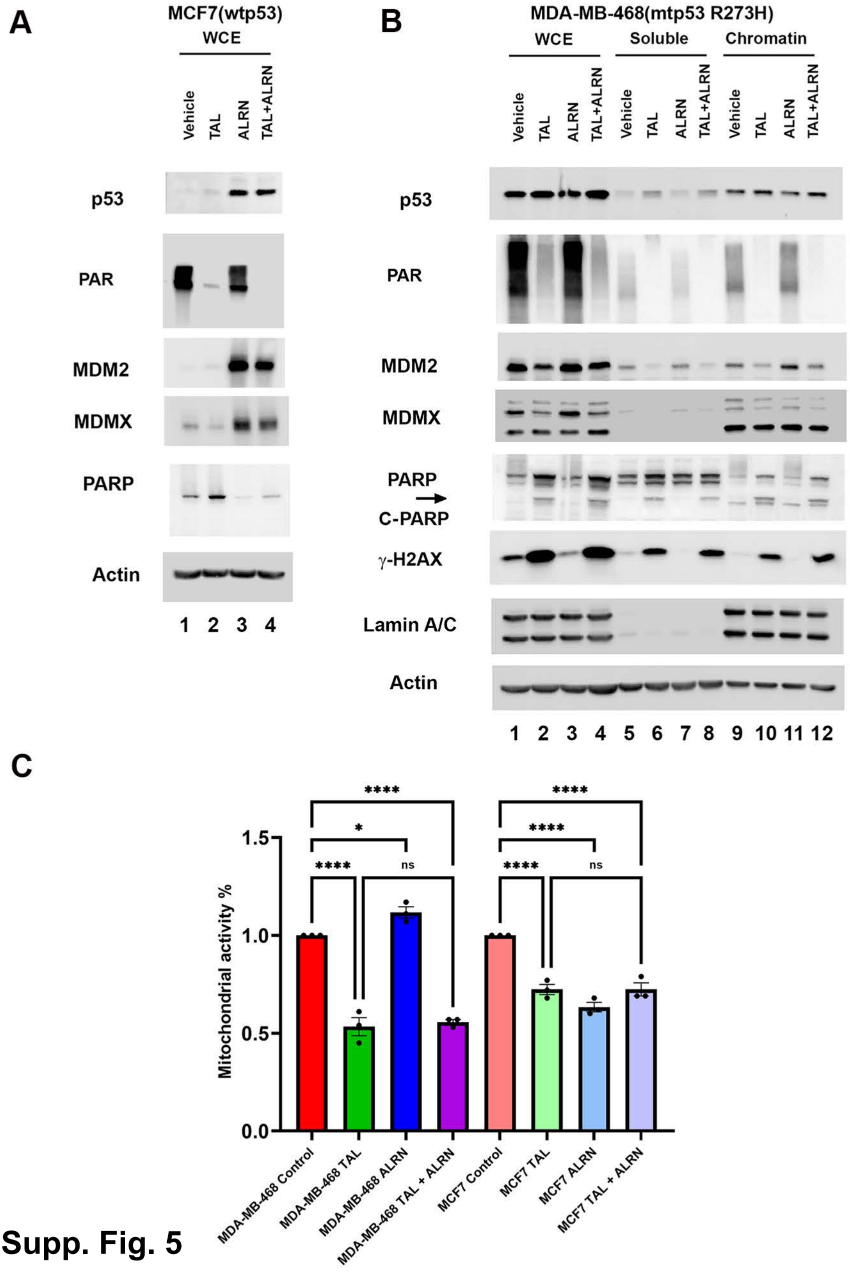

A
MCF7(wtp53)
WCE
Vehicle
TAL
ALRN
TAL+ALRN
p53
PAR
MDM2
MDMX
PARP
Actin
1 2 3 4
B
MDA-MB-468(mtp53 R273H)
WCE
Soluble
Chromatin
Vehicle
TAL
ALRN
TAL+ALRN
p53
PAR
MDM2
MDMX
PARP
C-PARP
γ-H2AX
Lamin A/C
Actin
1 2 3 4 5 6 7 8 9 10 11 12
C
Mitochondrial activity %
0.0
0.5
1.0
1.5
****
*
****
ns
ns
MDA-MB-468 Control
MDA-MB-468 TAL
MDA-MB-468 ALRN
MDA-MB-468 TAL + ALRN
MCF7 Control
MCF7 TAL
MCF7 ALRN
MCF7 TAL + ALRN

Supp. Fig. 5

D

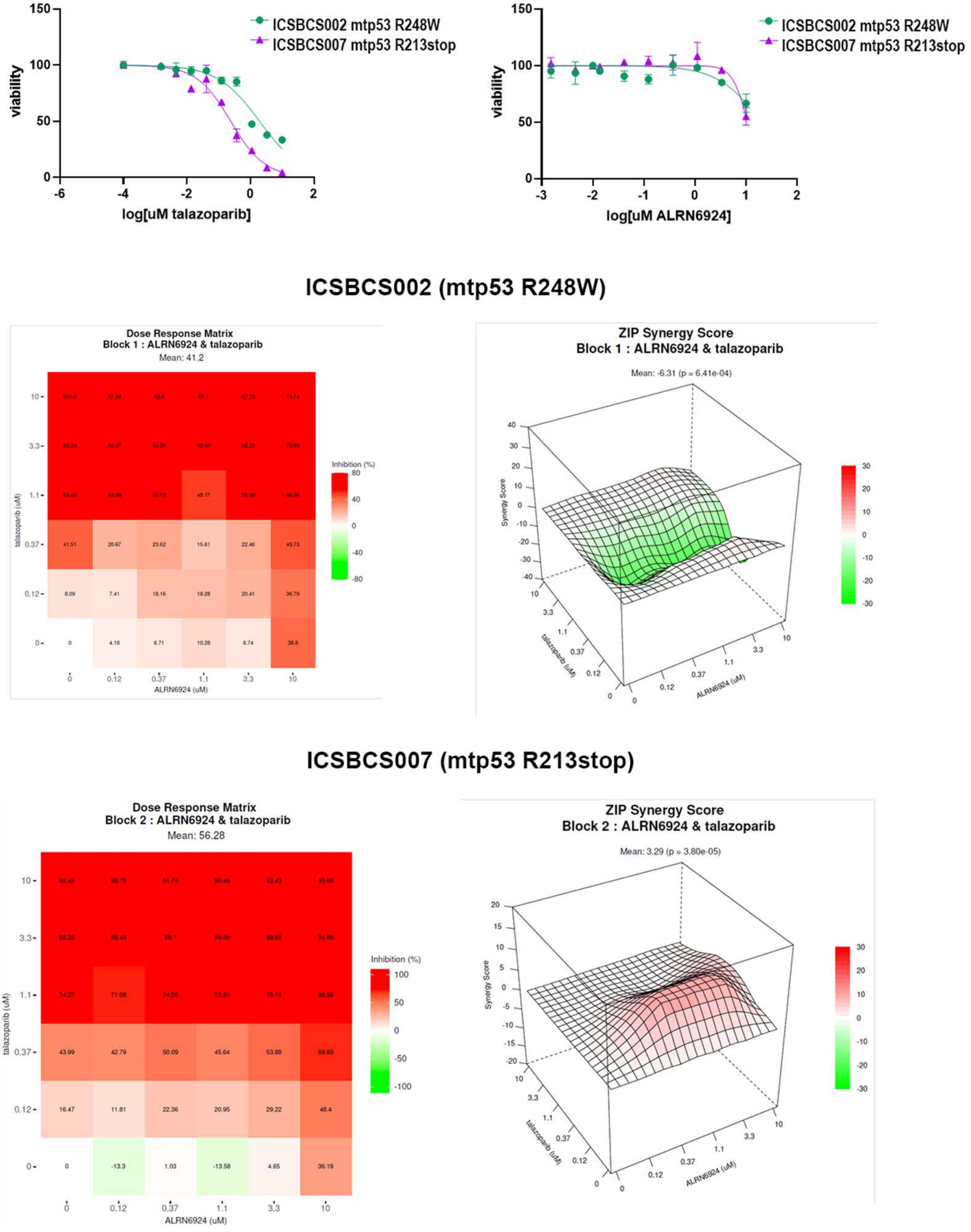

150
100
50
0
viability
-6
-4
-2
0
2
log[uM talazoparib]
ICSBCS002 mtp53 R248W
ICSBCS007 mtp53 R213stop
-3
-2
-1
1
log[uM ALRN6924]
ICSBCS002 (mtp53 R248W)
Dose Response Matrix
Block 1 : ALRN6924 & talazoparib
Mean: 41.2
41.51 20.67 23.62 15.61 22.46 43.73
8.09 7.41 18.16 18.28 20.41 36.79
0 4.18 6.71 10.28 6.74 39.8
talazoparib (uM)
ALRN6924 (uM)
0 0.12 0.37 1.1 3.3 10
Inhibition (%)
ZIP Synergy Score
Block 1 : ALRN6924 & talazoparib
Mean: -6.31 (p = 6.41e-04)
Synergy Score
ICSBCS007 (mtp53 R213stop)
Dose Response Matrix
Block 2 : ALRN6924 & talazoparib
Mean: 56.28
43.99 42.79 50.09 45.64 53.88 69.83
16.47 11.81 22.36 20.95 29.22 48.4
0 -13.3 1.03 -13.58 4.65 36.19
Inhibition (%)
ZIP Synergy Score
Block 2 : ALRN6924 & talazoparib
Mean: 3.29 (p = 3.80e-05)

Supp. Fig. 5
